# A study of local guidelines for use of an Early Warning Score System to identify patients in need of treatment in the Intensive Care Unit

**DOI:** 10.1186/1757-7241-21-S2-A14

**Published:** 2013-09-09

**Authors:** Jesper Kørup Jensen, Dorthe Hellemann, Gustav From

**Affiliations:** 1 Emergency Department, Slagelse Sygehus, Denmark; 2 Department of Anesthesiology, Slagelse Sygehus, Denmark

## Background

Scientific studies suggests that transfer delays from the Emergency Department(ED) to the Intensive Care Unit(ICU) increases mortality and morbidity.

A modified Standardized Early Warning Scoring System called Basal Observations Score(BOS) is used in Region Sjælland to monitor vital signs of in-hospital patients. Respiratory rate, peripheral O2-saturation, systolic blood pressure, heart rate, Glasgow Coma Scale, and urinary output are included. BOS is documented on special paper forms, and on electronic white boards. The clinical guidelines for the ED states that patients with BOS>5 should preferably be treated in the ICU.

The aim with this study was to evaluate the guidelines and the accordance between guidelines, and clinical practice.

## Methods

A retrospective cohort study was conducted, including all patients admitted to the ED with BOS≥5 on the electronic white board, from 1/5 to 16/10 2012. All journal entries from the ED were audited in order to validate BOS, and carry out a descriptive analysis of specified endpoints.

## Results

134 patients were included. 15 of these were not considered candidates for ICU admission by ED clinicians. 89 had BOS>5. There was no significant difference between the mortality (p>0,25), length of admission (p=0,134), or proportion of transfers to ICU between BOS=5 and BOS>5 (p>0,5).

15 patients were assessed by anesthesiologists (12,6%, 95%CI=6,64-18,57). 11 of these were transferred to the ICU (73,33%, 95%CI=50,95-95,71). The average length of stay in the ED was 237 minutes (95%CI=88-386).

## Conclusion

Fewer requests, for assessment by an anesthesiologist, were made than expected. Once assessed, the majority of patients were transferred to the ICU, suggesting that clinical practice in the ED was not in compliance with guidelines. Further studies are needed to clarify how the use of an anesthesiologist to optimize treatment, and evaluate transfer to ICU, is implemented.

Patients transferred to the ICU spent an average of almost 4 hours in the ED.

There was no difference between endpoints between patients with BOS=5 and BOS>5, and no significant difference in the time spent in the ED between the two groups. The conclusion is that these groups should be considered equal candidates for ICU admission in the clinical guidelines.

